# Vaccination

**Published:** 1871-09

**Authors:** 


### VACCINATION.

Small-pox is such a loathsome disease, and so fatal, that every man owes it to society to do what he can to prevent himself from taking it; because in having it he endangers all with whom he may come in contact. The following suggestions should be kept in remembrance in every household: —

Infants should be vaccinated within a month of birth.

This vaccination should be repeated at the age of fourteen.

The vaccine matter should be fresh, as the longer it is kept, the less efficacious does it become.

Scientific men have come to the conclusion that no other disease is communicated by the process of vaccination; the ground for that belief is, that in the experience of a number of eminent practitioners no such fact has ever been observed.

In some cases, "rashes" of various kinds follow vaccination, but the evidence is that such rashes are taints of blood or disease, which the influence, the power of the vaccination drives out of the system, and leaves it healthier than it ever was before.

In order to be more perfectly certain that any disease which a person may have had may not be communicated by the vaccine matter taken from his arm, the only precaution needed is to guard against any blood being taken up from the sore when the vaccine matter is abstracted from it.

The first vaccine matter was taken from a cow, the Latin name for which is *vaccina*, and there is reason to believe that in the course of years this matter has lost somewhat of its efficacy.

Some years ago, it was suggested in these pages that this matter ought to be renewed. An intelligent gentleman, a philanthropist of New England, makes it his business to keep cows on hand all the time, from which pure matter can be had. Physicians everywhere should make a note of this, and obtain some of the matter, fresh, every year. This gentleman's address is not known, or it would be given here.

